# Assessment of potato peel and agro-forestry biochars supplementation on in vitro ruminal fermentation

**DOI:** 10.7717/peerj.9488

**Published:** 2020-07-28

**Authors:** Ana R.F. Rodrigues, Margarida R.G. Maia, Ana R.J. Cabrita, Hugo M. Oliveira, Maria Bernardo, Nuno Lapa, Isabel Fonseca, Henrique Trindade, José L. Pereira, António J.M. Fonseca

**Affiliations:** 1 REQUIMTE, LAQV, ICBAS, Instituto de Ciências Biomédicas Abel Salazar, Universidade do Porto, Porto, Portugal; 2 INL, International Iberian Nanotechnology Laboratory, Braga, Portugal; 3 REQUIMTE, LAQV, Faculdade de Ciências e Tecnologia, Universidade Nova de Lisboa, Caparica, Portugal; 4 CITAB, Universidade de Trás-os-Montes e Alto Douro, Vila Real, Portugal; 5 ESAV, Instituto Politécnico de Viseu, Viseu, Portugal

**Keywords:** Agro-forestry biochar, Supplementation level, Rumen fermentation, Potato peel biochar, Enteric methane

## Abstract

**Background:**

The awareness of environmental and socio-economic impacts caused by greenhouse gas emissions from the livestock sector leverages the adoption of strategies to counteract it. Feed supplements can play an important role in the reduction of the main greenhouse gas produced by ruminants—methane (CH_4_). In this context, this study aims to assess the effect of two biochar sources and inclusion levels on rumen fermentation parameters *in vitro*.

**Methods:**

Two sources of biochar (agro-forestry residues, AFB, and potato peel, PPB) were added at two levels (5 and 10%, dry matter (DM) basis) to two basal substrates (haylage and corn silage) and incubated 24-h with rumen inocula to assess the effects on CH_4_ production and main rumen fermentation parameters *in vitro*.

**Results:**

AFB and PPB were obtained at different carbonization conditions resulting in different apparent surface areas, ash content, pH at the point of zero charge (pHpzc), and elemental analysis. Relative to control (0% biochar), biochar supplementation kept unaffected total gas production and yield (mL and mL/g DM, *p* = 0.140 and *p* = 0.240, respectively) and fermentation pH (*p* = 0.666), increased CH_4_production and yield (mL and mL/g DM, respectively, *p* = 0.001) and ammonia-N (NH_3_-N, *p* = 0.040), and decreased total volatile fatty acids (VFA) production (*p* < 0.001) and H_2_ generated and consumed (*p* ≤ 0.001). Biochar sources and inclusion levels had no negative effect on most of the fermentation parameters and efficiency. Acetic:propionic acid ratio (*p* = 0.048) and H_2_ consumed (*p* = 0.019) were lower with AFB inclusion when compared to PPB. Biochar inclusion at 10% reduced H_2_ consumed (*p* < 0.001) and tended to reduce total gas production (*p* = 0.055). Total VFA production (*p* = 0.019), acetic acid proportion (*p* = 0.011) and H_2_ generated (*p* = 0.048) were the lowest with AFB supplemented at 10%, no differences being observed among the other treatments. The basal substrate affected most fermentation parameters independently of biochar source and level used.

**Discussion:**

Biochar supplementation increased NH_3_-N content, *iso*-butyric, *iso*-valeric and valeric acid proportions, and decreased VFA production suggesting a reduced energy supply for microbial growth, higher proteolysis and deamination of substrate N, and a decrease of NH_3_-N incorporation into microbial protein. No interaction was found between substrate and biochar source or level on any of the parameters measured. Although AFB and PPB had different textural and compositional characteristics, their effects on the rumen fermentation parameters were similar, the only observed effects being due to AFB included at 10%. Biochar supplementation promoted CH_4_ production regardless of the source and inclusion level, suggesting that there may be other effects beyond biomass and temperature of production of biochar, highlighting the need to consider other characteristics to better identify the mechanism by which biochar may influence CH_4_ production.

## Introduction

A multidisciplinary effort for developing efficient approaches for greenhouse gas emissions mitigation emerged from the growing concerns on global climate change. The livestock sector represents 14.5% of carbon dioxide equivalent of the total anthropogenic emissions (Gerber et al., 2013), ruminants being estimated to be responsible for *c.a.* 80% of these emissions. In ruminant production, independently of the species, methane (CH_4_) comprises the largest source of greenhouse gas emissions, with enteric fermentation being responsible for 90% of CH_4_ production and manure for the remaining 10% (Opio et al., 2013). Enteric CH_4_ production occurs by the reduction of carbon dioxide with hydrogen by the rumen archaea population (Hill et al., 2016). Methanogenesis not only contributes to the impact of ruminant production on climate change but also represents 2–12% of energy lost by the animal (Johnson & Johnson, 1995). Hence, strategies to reduce CH_4_ enteric production may lead to a win-win situation to the environment and economy of the livestock sector.

Biochar, a carbon-rich product obtained by thermal decomposition of biomass under a limited supply of oxygen at high temperatures (typically lower than 700 °C), has been successfully used to amend soils and to increase their nutrient availability beyond a fertilizer effect (Lehmann & Joseph, 2010). Due to its carbon sequestration ability (Lehmann & Joseph, 2010), biochar can contribute to mitigate the emissions of CH_4_ from soils (Jeffery et al., 2016), while promoting soil structure and crop production (Liu et al., 2013). These results triggered a growing interest in the dietary supplementation of biochar as a potential strategy to reduce CH_4_ formed during the rumen fermentation. The mechanism behind the decrease of gas and CH_4_ productions through biochar supplementation is still unclear, but possible explanations include: (i) gas adsorption (Hansen, Storm & Sell, 2012), (ii) a direct toxic effect on methanogens (Leng, Inthapanya & Preston, 2012a), (iii) substrate for the biofilms that provide a favorable habitat for the synergic methanogenic-methanotrophic interactions (Leng, 2014; Leng, Inthapanya & Preston, 2012a), and (iv) electron mediators in biochemical redox reactions, enabling direct interspecies electron transfer (Chen et al., 2014; Schmidt et al., 2019).

Furthermore, the oral administration of biochar to ruminant animals may have additional advantages, namely in the (1) sequestration of the GHG emitted from feces and manure, in the (2) reduction of water and air pollution caused by the direct application of manure to soil (Blackwell, Riethmuller & Collins, 2009), and in the (3) improvement of nutrient intake efficacy and of overall animal health (Toth et al., 2016). Notwithstanding these facts, few studies have evaluated the effect of biochar on rumen fermentation. The current studies suggest that effects on rumen fermentation depend on biochar source and size (McFarlane et al., 2017), albeit effects on CH_4_ are contradictory. Indeed, biochar supplementation was reported to reduce (Calvelo Pereira et al., 2014; Vongsamphanh et al., 2015) or kept unaffected (Calvelo Pereira et al., 2014; Hansen, Storm & Sell, 2012) enteric CH_4_ production. Therefore, more research is needed to determine the role and potential benefits of different biochar sources and inclusion levels on rumen fermentation parameters. In addition, the putative interaction of biochar sources and inclusion levels with the basal substrates should be examined. Indeed, Castro-Montoya et al. (2012) reported interactions between methane mitigation additives and basal substrates on CH_4_ and VFA productions.

In this context, this study expands the knowledge about the role of biochar in rumen fermentation. It assesses, for the first time, the effect of agro-forestry (AFB) and potato peel (PPB) biochars, included at two inclusion levels (5, and 10% dry matter, DM, basis) on rumen fermentation in vitro (24-h batch) incubations. Basal substrate source (haylage, HS, and corn silage, CS) on biochar supplementation effects were also evaluated.

## Materials & Methods

Effects of biochar supplementation on the rumen fermentation parameters were evaluated in 24-h in vitro incubations, according to a factorial design with 2 biochar sources (AFB and PPB), 2 biochar inclusion levels (5% or 10%, DM basis), and 2 basal substrates (HS and CS).

### Biochars and basal substrates

AFB and PPB were produced from by-products of the Portuguese food industry and forestry sector.

AFB was produced through the slow pyrolysis of agro-forestry biomass wastes (vineyard pruning residues and acacia wood) at a temperature of 600 C for c.a. 6 h. PPB was produced by lab-scale pyrolysis conducted in a quartz reactor, which was placed in a vertical furnace; potato peel waste from an appetizer industry was heated up to 500 °C at 10 °C/min, and the temperature held for 2 h under a N_2_ flow of 150 mL/min. Conditions chose for AFB and PPB production were based on previous thermogravimetric analysis (TGA) and pyrolysis experiments that showed thermostability of these materials and therefore a reasonable biochar amount.

HS and CS were used as basal substrates in the in vitro incubations as well as dietary ingredients of the rumen inocula donor diets. Basal substrates were dried in a forced-air oven, for 48 h at 65 °C, ground to pass through a 1-mm screen and stored at room temperature until incubation.

### Biochars characterization

The textural properties of biochars such as apparent surface area, total pore and micropore volumes were evaluated by the adsorption isotherm of N_2_ at −196 °C obtained in an Accelerated Surface Area and Porosimetry System (ASAP 2010, Micromeritics Instruments Corp. Norcross, GA). Adsorption measurements were made after outgassing the sample overnight under vacuum at 150 °C. The adsorption data were used to calculate the apparent surface area (S_BET_) through the BET equation (Brunauer, Emmett & Teller, 1938). The total pore volume (V_total_) was determined by the amount of N_2_ adsorbed at the relative pressure p/p_0_ = 0.99. The micropore volume (V_micro_) was evaluated by the t-plot method, and the mesopore volume (V_meso_) was determined by the difference between V_total_ and V_micro_.

The surface chemistry of the biochars was characterized by the determination of the pH at the point of zero charge (pH_PZC_), based on the following methodology: 0.1 g of biochar was mixed with 20 mL of 0.1 M NaCl solution in different flasks. The pH of NaCl solution was adjusted with the addition of 0.1 M solutions of NaOH or HCl to the desired initial pH value (between 2 and 12) in different flasks. Biochars were then added to each flask, the mixtures were shaken for 24 h to reach equilibrium, and the final pH measured (MicropH 2001 pH meter, Crison Instruments SA, Spain). The pH_PZC_ value corresponds to the plateau of the curve pH_final_ *vs* pH_initial_.

Elemental analysis to determine C, H, N, and S contents in biochars was performed on an Elemental Analyzer Thermo Finnigan (CE Instruments, model Flash EA 1112 CHNS series) based on sample combustion dynamics. Ash content was also determined at 750 °C as specified in ASTM D 1762 standard (wood charcoal).

### Rumen inocula donor animals and diets

Two adult Holstein cows, dry and not pregnant, fitted with rumen cannula (10 cm diameter; Bar Diamond Inc., Parma, ID), housed at the Vairão Agricultural Campus of Abel Salazar Biomedical Sciences Institute, University of Porto (ICBAS-UP, Vila do Conde, Portugal), were used as rumen inocula donors. Care and management of cows followed the European Union good animal practices (Directive 2010/63/EU). Animal procedures and methodologies were reviewed and approved by the Animal Ethics Committee of ICBAS-UP, licensed by the Portuguese Directorate-General of Food and Veterinary Medicine (permit #FT2014DGV 046412 ICB), and conducted by trained scientists (FELASA category C).

To achieve true replication of the rumen inocula, cows were fed three total mixed rations (TMR) in three experimental periods of 30 days each. In the first period, one cow was fed a HS-based TMR and the other cow a CS-based TMR. Diets were exchanged between cows in the second period and, in the third period, one cow was fed a TMR based on CS and HS (Table 1). Cows were fed twice a day, at 09:30 h and 17:30 h, the daily amount of TMR being equally divided between meals. Orts were collected daily. Cows had *ad libitum* access to fresh water. After the 30 days adaptation of each experimental period, contents from all quadrants of the rumen were collected to a pre-warmed (39 °C) thermos container and transported to the laboratory within 60 min.

**Table 1:** Ingredient and chemical composition (g/kg dry matter) of the animal diets and basal substrates used in the in vitro incubations.

	**Cows diets**			**Basal substrates**	
	**TMR1**	**TMR2**	**TMR3**	**Haylage**	**Corn silage**
**Ingredient composition, g/kg DM**					
Haylage	723	–	491		
Corn silage	–	452	242		
Wheat straw	–	224	–		
Concentrate	277	324	267		
**Chemical composition, g/kg DM**					
Ash	80.1	56.6	69.4	72.7	34.5
Organic matter	920	943	931	927	965
Crude protein	104	116	119	55.3	77.4
Neutral detergent fiber	591	454	509	704	392
Ether extract	21.1	28.2	27.2	10.6	48.5
Starch	51.6	227	139	n.d.	369

**Notes.**

DM dry matter n.d. not determined

### Chemical analysis

Feed ingredients of the experimental diets offered to the rumen inocula donors were collected in each experimental period for chemical analysis, including the HS and CS used as basal substrates in in vitro incubations. Thus, diets and basal substrates were analyzed for DM (method 934.01, AOAC, 1990), ash (method 942.05, AOAC, 1990), Kjeldahl N (method 954.01, AOAC, 1990), ether extract (method 920.39, AOAC, 1990) and neutral detergent fiber (NDF, with *α*-amylase and without sodium sulfite, and expressed exclusive of residual ash; Robertson & Van Soest, 1981; Van Soest, Robertson & Lewis, 1991). Crude protein (CP) was calculated as Kjeldahl N × 6.25. The starch content of CS and concentrate used in the experimental diets was determined on finely (0.5-mm) ground samples (Salomonsson, Theander & Westerlund, 1984). All chemical analyses were run in duplicate.

### Rumen in vitro incubations

The ruminal contents of each animal were individually homogenized and strained through four layers of linen cloth. One part of each strained rumen fluid was diluted anaerobically with four parts of a buffer solution (Mould et al., 2005). Treatments comprised two basal substrates (HS and CS) supplemented with two biochar sources (AFB and PPB) at two inclusion levels (5 and 10%, DM basis). Two hundred and fifty milligrams DM of treatments (237.5 or 225.0 mg substrate and 12.5 or 25.0 mg biochar, respectively, for 5 and 10% inclusion) or controls (basal substrate without substrate) were weighted, in 125 mL serum bottles (Sigma-Aldrich Inc., St. Louis, MO). Blank samples (without substrate or biochar) were also included. Fifty milliliters of buffered rumen fluid were added to each serum bottles (treatments, control and blanks). Anaerobiosis was achieved by flushing the bottles with O_2_-free CO_2_, and immediately sealing them with butyl rubber stoppers and aluminum caps. All treatments, controls, and blanks were incubated in triplicate (laboratory replicates), per incubation run and per experimental period, in a water bath at 39 °C. After 24 h of incubation, the fermentation was stopped by cooling the serum bottles into an ice water bath.

One incubation run of each individual inocula was carried out with 30 days interval.

### Sampling, analysis, and calculations

Bottles were stepwise warmed up to 25 °C, and the fermentation gas volume was measured with a pressure transducer (Theodorou et al., 1994). A gas sample was collected with a gas-tight syringe (SGE international PTY Ltd, Australia), and CH_4_ analyzed by gas chromatography, using a GC-4000A (East & West Analytical Instruments, Inc, Beijing, China) equipped with a Shincarbon ST 100/120 micropacked column (Restek Corporation, Bellefonte, PA) and a thermal conductivity detector (Maia et al., 2016). Total gas volume of samples was corrected for gas volume of blanks. CH_4_ production was estimated from the CH_4_ proportion in the gas sample corrected for blanks, the headspace volume of the incubation vessel and the ideal gas laws (Lopez & Newbold, 2007). Gas and CH_4_ yields were calculated as production volume (mL) per g of total (substrate and biochar) DM incubated.

After gas sampling, fermentation pH was immediately measured and fermentation media sampled for volatile fatty acids (VFA) and ammonia-N (NH_3_-N) analysis. VFA were analyzed by gas chromatography using a Shimadzu GC-2010 Plus (Shimadzu Corporation, Kyoto, Japan) equipped with a capillary column (HP-FFAP, 30 m × 0.25 mm × 0.25 µm; Agilent Technologies, Santa Clara, CA), and a flame ionization detector. The individual VFA were identified and quantified as described by Maia et al. (2019). NH_3_-N was analyzed by steam-distillation and the N content determined by titration (method 954.01, AOAC, 1990). Total VFA production and NH_3_-N content were corrected for blanks.

Individual VFA and CH_4_(molar basis) were used in stoichiometric models to predict the reducing equivalents generated and the reducing equivalents consumed (expressed as µmol H_2_/mL fermentation media; Moss, Jouany & Newbold, 2000). Reducing equivalents generated were estimated as 2 acetic acid + 1 propionic acid + 4 butyric acid + 2 valeric acid + 2 *iso*-valeric acid, and reducing equivalents consumed as 2 propionic acid + 2 butyric acid + 1 valeric acid + 4 CH_4_ (Demeyer, 1991). Recovery (%) was calculated as reducing equivalents consumed/reducing equivalents generated × 100 (Demeyer, 1991). Fermentation efficiency was calculated as (0.62 acetic acid + 1.09 propionic acid + 0.78 × butyric acid)/(acetic acid + propionic acid + butyric acid) × 100 and is based on the heats of combustion of glucose in the respective VFA (Chalupa, 1977).

### Statistical analysis

Laboratory replicates were averaged before statistical analysis.

Data on the rumen fermentation parameters were analyzed using the MIXED procedure of SAS (2002; version 9.1, SAS Institute Inc., Carry, NC). The model included the fixed effects of biochar source (AFB and PPB) and level (5 and 10%), basal substrate (HS and CS), the interactions between biochar source and level, between biochar source and basal substrate and among biochar source, biochar level and basal substrate, the random effect of incubation run, and the random residual error. Differences among the means were identified using Tukey’s multiple comparisons. Effects were considered significant when *p* ≤ 0.05 and a trend when 0.05 <*p* ≤ 0.10. When the interaction was not significant, nor a tendency was observed (*p* > 0.10), it was removed from the model.

Student’s *t*-test was used to compare the overall mean value of biochar supplementation with the overall mean value of control (basal substrate without biochar).

## Results

In the present study, the interactions among biochar source, biochar level, and basal substrate and between biochar source and basal substrate were not significant nor a tendency was observed. Therefore, our study focused on the main effects of biochar source, biochar level and basal substrate and on the biochar source and biochar level interaction on gas production, methane production, and rumen fermentation parameters, after 24-h in vitro incubation.

### Chemical composition of substrates and biochars

The TMR offered to the cows’ donors of rumen inocula had similar protein content and mainly differed on NDF and starch contents (Table 1). Regarding the basal substrates, NDF and CP contents were respectively 704 and 55.3 g/kg DM for HS and 392 and 77.4 g/kg DM for CS. Starch content of CS was 369 g/kg DM (Table 1).

AFB and PPB characterization are presented in Table 2. Apparent surface area (S_BET_) was 94 and <5 m^2^/g for AFB and PPB, respectively. Ash content was lower for AFB than for PPB (8.62 and 21.9 w/w %, respectively). The pH_PZC_ value was 8.5 for AFB and 10.0 for PPB.

**Table 2:** Characterization of the agro-forestry biochar and the potato peel biochar used in the in vitro incubations.

	**Agro-forestry biochar**	**Potato peel biochar**
Carbonization conditions	600 °C, around 6 h	Heating rate of 10 °C/min until 500 °C, reaction time of 2 h, N_2_ flow of 150 mL/min. Yields around 30%
Surface area (S_BET_), m^2^/g	94	<5
Total pore volume, cm^3^/g	0.05	n.q.
Micropore volume, cm^3^/g	0.03	n.q.
Mesopore volume, cm^3^/g	0.02	n.q.
pH_PZC_	8.5	10.0
Ash content, % w/w	8.62	21.9
Elemental analysis, % w/w (as received basis)	*C* = 85.09; *H* = 1.81; *N* = 0.35; *S* = 0.00; *O* = 4.13	*C* = 65.74; *H* = 0.98; *N* = 1.86; *S* = 0.00; *O* = 9.52

**Notes.**

n.q. not quantifiable

### Basal substrates effects

Basal substrates had a significant effect on the rumen fermentation parameters from in vitro 24-h batch incubation (Table 3). CS decreased (*p* < 0.001) pH and acetic acid, *iso*-butyric, and valeric proportions, and increased (*p* < 0.05) total gas production and yield, CH_4_ production and yield, total VFA production, butyric acid and caproic acid proportions, H_2_ generated and consumed, recovery and fermentation efficiency. NH_3_-N, propionic acid, *iso*-valeric acid and *iso*-caproic acid proportions and acetic:propionic ratio were unaffected (*p* > 0.05).

**Table 3:** Effect of control diet (basal substrate without biochar), biochar source and inclusion level, and substrate on the rumen fermentation parameters from in vitro 24-h batch incubation.

			**Agro-forestry biochar**		**Potato peel biochar**			***p***			**Substrate**			
	**Control**	***p***	**5%**	**10%**	**5%**	**10%**	**SEM**	**S**	**L**	**SxL**	**Haylage**	**Corn silage**	**SEM**	***p***
Total gas, mL	52.8	0.140	52.6	50.0	51.8	51.0	1.28	0.941	0.055	0.299	45.0	57.7	1.12	<0.001
Total gas, mL/g DM	221	0.240	222	211	218	214	5.4	0.999	0.555	0.311	190	242	4.71	<0.001
Methane, mL	4.02	0.001	4.67	4.71	4.71	4.43	0.465	0.541	0.535	0.411	3.75	5.51	0.445	<0.001
Methane, mL/g DM	16.9	0.001	19.7	19.8	19.8	18.6	1.96	0.522	0.534	0.403	15.9	23.1	1.88	<0.001
pH	6.36	0.666	6.36	6.37	6.36	6.36	0.014	0.980	0.349	0.324	6.39	6.33	0.013	<0.001
NH_3_-N, mg/g DM	15.1	0.040	16.7	16.9	16.3	18.0	2.54	0.664	0.218	0.402	17.1	16.9	2.48	0.840
Total VFA, mmol/g DM	11.5	<0.001	11.0^b^	10.2^a^	11.2^b^	10.9^b^	0.82	0.001	<0.001	0.019	10.3	11.4	0.82	<0.001
Acetic acid, %	65.0	0.306	65.0^b^	64.2^a^	65.0^b^	65.2^b^	0.51	0.025	0.115	0.011	66.1	63.6	0.49	<0.001
Propionic acid, %	18.6	0.831	18.3	18.5	18.3	18.2	0.87	0.656	0.732	0.637	18.5	18.1	0.84	0.174
*Iso*-butyric acid, %	1.20	<0.001	1.22^a^	1.29^b^	1.23^a^	1.23^a^	0.010	<0.001	<0.001	<0.001	1.26	1.22	0.008	<0.001
Butyric acid, %	11.2	0.670	11.4	11.7	11.4	11.3	0.56	0.209	0.542	0.123	9.98	12.9	0.56	<0.001
*Iso*-valeric acid, %	2.21	0.002	2.26	2.39	2.27	2.27	0.075	0.243	0.195	0.194	2.30	2.30	0.068	0.881
Valeric acid, %	1.43	0.048	1.43^ab^	1.48^b^	1.43^ab^	1.42^a^	0.042	0.027	0.216	0.014	1.43	1.38	0.041	<0.001
*Iso*-caproic acid, %	0.0152	0.438	0.0146	0.0232	0.0145	0.0143	0.00428	0.299	0.329	0.306	0.015	0.019	0.0030	0.374
Caproic acid, %	0.384	0.980	0.378	0.389	0.379	0.369	0.0306	0.704	0.969	0.669	0.329	0.428	0.0251	<0.001
Enanthic acid, %	0.0209	0.937	0.0180	0.0195	0.0183	0.0216	0.00484	0.704	0.969	0.699	0.017	0.022	0.0046	0.052
Acetic:propionic acid ratio	3.56	0.866	3.61	3.52	3.61	3.62	0.194	0.048	0.592	0.479	3.59	3.59	0.186	0.998
H_2_ generated, mmol/L	110	<0.001	106^b^	98.0^a^	107^b^	104^b^	7.97	0.003	<0.001	0.048	96.2	111	7.92	<0.001
H_2_ consumed, mmol/L	33.7	0.001	32.5	30.6	32.9	31.9	1.85	0.019	<0.001	0.165	28.9	35.1	1.84	<0.001
Recovery, %	31.0	0.419	30.9	31.4	30.9	30.7	0.75	0.234	0.532	0.270	30.2	31.8	0.72	<0.001
Fermentation efficiency, %	73.1	0.978	73.0	73.2	73.0	72.9	0.36	0.391	0.593	0.319	72.9	73.2	0.34	0.049

**Notes.**

DM dry matter NH_3_-N ammonia-N VFA volatile fatty acids SEM standard error of the mean S biochar source L biochar inclusion level SxL interaction between biochar source and biochar inclusion level

a,b,c,d Means in the same row followed by different letters differ (*p* < 0.05).

### Biochar supplementation effects

When compared to control, biochar supplementation kept unaffected (*p* > 0.05) total gas production and yield, pH, acetic acid, propionic acid, butyric acid, *iso*-caproic acid, caproic acid, and enanthic acid proportions, acetic:propionic ratio, recovery, and fermentation efficiency (Table 3).

Biochar supplementation increased CH_4_ production and yield (*p* = 0.001), NH_3_-N (*p* = 0.040), *iso*-butyric acid (*p* < 0.001), *iso*-valeric acid (*p* = 0.002), and valeric acid (*p* = 0.048) proportions. Conversely, biochar supplementation decreased total VFA production (*p* < 0.001), as well H_2_ generated (*p* < 0.001) and H_2_ consumed (*p* = 0.001, Table 3).

### Biochar source and biochar inclusion level effects

The effects of biochar source and biochar inclusion level on the rumen fermentation parameters were sparse. Biochar source only affected acetic:propionic acid ratio (*p* = 0.048) and H_2_ consumed (*p* = 0.019), with PPB promoting both parameters. Biochar level impacted in the H_2_ consumed (*p* < 0.001) and total gas production (*p* = 0.055). All the other parameters were kept at similar levels for both biochar source and level (*p* > 0.05, Table 3).

Total VFA production (*p* = 0.019), acetic acid proportion (*p* = 0.011) and H_2_ generated (*p* = 0.048) were lowest with AFB supplemented at 10%, no differences being observed among the other treatments. Conversely, AFB supplemented at 10% led to the highest *iso*-butyric acid proportion (*p* < 0.001, Table 3).

## Discussion

Several strategies have been proposed to mitigate enteric CH_4_ emissions from ruminants, the inclusion of feed supplements being one of the most common. In this research work, AFB and PPB were evaluated as dietary supplements for their effects on overall rumen fermentation parameters in 24-h in vitro batch incubations. The batch incubation technique was chosen since it is a simple method to evaluate the potential effect of novel feed ingredients and additives on the fermentation parameters prior to in vivo studies (Klop et al., 2016). The reuse of industrial and forestry by-products into animal feed contribute to a more sustainable ruminant production sector pursuing a circular economy model.

### Effect of basal substrate on rumen fermentation parameters

Basal substrates had a marked effect on the rumen fermentation parameters from in vitro 24-h batch incubation, with CS leading to a lower pH and to a higher total VFA, gas and CH_4_ productions, H_2_ generated and consumed, recovery and fermentation efficiency than HS. Overall, these results reflect a greater extent of fermentation of CS relatively to HS. In addition, CS promoted a lower acetic acid proportion but failed to promote propionic acid proportion. Microbial fermentation of feeds in the rumen cause several end-products with numerous functions in the host animal (Bergman, 1990). The profile of these fermentation end-products is largely dependent on the feed nutrient composition. Roughage-based diets are known to promote the production of acetic acid whereas starch-rich diets decrease acetic acid proportion, increase propionic acid proportion and reduce fiber digestibility through decreased pH (Hoover, 1986). Both acetic acid and butyric acid promote CH_4_ production, whereas propionic acid comprises a competitive pathway for H_2_ use in the rumen, thus decreasing CH_4_ production (Moss, Jouany & Newbold, 2000).

As acetic and butyric acids proportion were differently affected by substrates and that of propionic acid was unaffected, the promoted CH_4_ production observed with CS when compared to HS may be explained by the higher extent of fermentation with CS, as supported by the increased VFA production and H_2_ generated and consumed with this substrate. Results of CS on the rumen fermentation parameters herein reported strongly agree with those of Maia et al. (2016).

### Effect of biochar on methane and total gas production in vitro

In the present study, biochar supplementation increased CH_4_ production and yield, and kept total gas production and yield unaffected when compared with control.

Effects of biochar on CH_4_ production reported in literature are ambiguous, from neutral effect to an observed reduction of CH_4_ being reported in vitro (Calvelo Pereira et al., 2014; Hansen, Storm & Sell, 2012; Leng, Inthapanya & Preston, 2012a; Leng, Inthapanya & Preston, 2012b; Leng, Inthapanya & Preston, 2013; Terry et al., 2019; Vongkhamchanh, Inthapanya & Preston, 2015; Vongsamphanh et al., 2015) and in vivo (Leng, Preston & Inthapanya, 2012). Such differences may reflect the different biomass and temperatures used to prepare biochars, which also confound comparison among studies. Leng, Inthapanya & Preston (2012a) suggested that the positive effects observed on CH_4_ reduction are related to the high temperature (900 °C) used in biochar production. However, other studies reported a similar effect on CH_4_ production of biochars prepared at 350 °C and 550 °C (Calvelo Pereira et al., 2014), and even higher methanogenesis reduction with biochar prepared at 550 °C than at 700 °C (Cabeza et al., 2018). In our study, two biochar sources were used (AFB and PPB), prepared at two different temperatures (600 °C and 500 °C, respectively), yet the effects on CH_4_ production and yield were similar. Additionally, the biochars used had different surface areas (AFB, 94 m^2^/g; PPB, <5 m^2^/g), that may reflect its capacity to trap gases, and pH_PZC_ (pH at the point of zero charge), that measures the ionic state of functional groups which affect the adsorption capacity of biochar (Mondal et al., 2016). When compared to PPB, the higher S_BET_ of AFB (94 m^2^/g) may be related to the experimental pyrolysis conditions used to produce this biochar (higher temperature and residence time), allowing a higher devolatilization degree and porosity development. Indeed, Calvelo Pereira et al. (2014) found higher S_BET_ when biochars from pine chips and corn stover were obtained by slow pyrolysis at 550 °C than at 350 °C. Large surface to weight ratio (between 30 and 500 m^2^/g) was related to increased biofilm formation (Leng, Inthapanya & Preston, 2013), thus suggesting that AFB could promote more microbial film formation in the rumen than PPB. Nevertheless, considering the similar performance of both biochars, other factors than S_BET_ may play a major role in total gas and CH_4_ production and yield. Although AFB and PPB had different pH_PZC_ (8.5 and 10.0, respectively), both kept unchanged CH_4_ production and yield. Similarly, no effects of biochar source on CH_4_ were reported with alkaline biochars (pH >8; Calvelo Pereira et al., 2014; Hansen, Storm & Sell, 2012; Teoh et al., 2019) whereas acidic biochar (pH 4.8) lowered CH_4_ production and yield (Saleem et al., 2018).

Another possible explanation for the results obtained in the present study, comprises the use of rumen fluid, low in digesta particles, from cows not adapted to biochar. Indeed, the reduction of net CH_4_ production was found with rumen fluid from cows adapted to biochar Leng, Inthapanya & Preston (2012b). Multi-species biofilms have been described to establish on the external surface of the plant particles, also developing inside of the feed particles with the solubilization and utilization of the substrate (McAllister et al., 1994). These structures host the sequential degradation of complex structural and readily fermentable carbohydrates through several classes of microorganisms that complement each other. This sequential fermentation requires a low partial pressure of H_2_ controlled by methanogenesis (Leng, Inthapanya & Preston, 2012b). Inert materials, such as biochar, suspended in the fluid or associated with feed particles, will contribute to uptake the solubilized feed materials. This will also contribute to the diffusion of H_2_ from the digestion of feed particles by providing a high surface area (Leng, Inthapanya & Preston, 2012b). Biochar could thus create a suitable habitat for the association of microorganisms that will simultaneously allow a more efficient fermentation and promote CH_4_ oxidation by joining archaeas and methanotrophic consortia (Knittel & Boetius, 2009). Nevertheless, the biochar-microbe interaction is another key aspect that still needs further investigation to clarify its role not only in rumen fermentation but also in other similar anaerobic processes (Mumme et al., 2014).

### Effect of biochar on rumen fermentation in vitro

After 24-h incubation, biochar supplementation increased NH_3_-N content when compared to control. An increase in NH_3_-N could reflect higher proteolysis and deamination of substrate N or a decrease of NH_3_-N incorporation into microbial protein (Bach, Calsamiglia & Stern, 2005). The observed biochar effect could be due to both processes. Hence, effects of biochar on *iso*-butyric acid, *iso*-valeric acid and on valeric acid proportions suggest increased proteolysis and deamination, as these VFA are known to be originated from the microbial fermentation of valine, leucine, and proline, respectively (Andries et al., 1987). Additionally, the similar gas production and lower VFA production observed with biochar supplementation than with control suggest a reduced energy supply for microbial growth that can be related to lower microbial protein synthesis. Nevertheless, this rationale still needs to be confirmed by further research since protein metabolism is out of the scope of the present work.

Different effects of biochars sources on NH_3_-N have been reported in the literature. Some studies showed that the biochars’ ability to adsorb NH_3_-N is inversely related to pyrolysis temperature at which biochar was produced (Cabeza et al., 2018; Zheng et al., 2013), as well as the effect of biomass source on cation adsorption (Gai et al., 2014). Furthermore, ash content has been implicated in NH${}_{4}^{+}$-N sorption ability because when ash is washed off some of the exchangeable cations might be removed from the biochar surface (Gai et al., 2014; Zheng et al., 2013). Despite the different pyrolysis temperatures to produce AFB and PPB biochar (600 and 500 °C, respectively) and their significantly different ash content (8.62 and 21.9% w/w, respectively), no effects were observed between biochar source (AFB and PPB) and between level (5 and 10%) on NH_3_-N content. Therefore, although the sorption capability of NH_3_-N was not measured, the AFB and PPB biochars seem to have similar NH_3_-N sorption ability.

Compared to control, biochar supplementation decreased total VFA production and increased *iso*-butyric acid, *iso*-valeric acid, and valeric acid proportions after 24-h in vitro fermentation. The decreased VFA production is consistent with the lower fermentable OM content of the biochar treatments, as AFB and PPB replaced either 5 or 10% of total DM incubated. However, previous studies have reported biochar to either increase VFA production or kept it unaffected (Cabeza et al., 2018; Saleem et al., 2018; Teoh et al., 2019). Different effects on individual VFA profile related with biochar supplementation have also been described, including reduced propionic acid and butyric acid production (Cabeza et al., 2018), no effect on acetic (Calvelo Pereira et al., 2014) or on none of the individual VFA (Teoh et al., 2019), and increased acetic acid, propionic acid and branched-chain fatty acids (Saleem et al., 2018). The contrasting results obtained in previous studies may be related to different biochar characteristics and inclusion levels that could influence the rumen microbial ecosystem and the profile of the fermentation end-products. Biochars from different biomasses have been supplemented from 0.5% (Saleem et al., 2018; Terry et al., 2019) up to 18.6% (Calvelo Pereira et al., 2014), DM basis, with a broad range of effects on in vitro rumen fermentation parameters. In our study, an average (5%) and a high (10%) biochar inclusion level were assessed, but the lower fermentable OM content, particularly at the highest inclusion level, may have contributed to the results herein presented. This highlights the importance of a full characterization of the biochar properties and methodological studies in order to better identify the factors by which biochar may affect the rumen fermentation parameters in both in vitro and in vivo.

No interaction was found between substrate and biochar source nor between substrate and biochar level on any of the parameters measured, agreeing with the results of Saleem et al. (2018) and Teoh et al. (2019). Conversely, McFarlane et al. (2017) suggested an improved fermentation when forages of intermediate quality but not of high quality, were supplemented with biochar. In the present study, both corn silage and haylage presented lower CP content and lower and similar NDF content, respectively than the substrate used by McFarlane et al. (2017; 9.8% CP and 70.4% NDF). On the other hand, the substrates used by Saleem et al. (2018) and Teoh et al. (2019) presented higher CP and lower NDF contents compared to the study of McFarlane et al. (2017). This inconsistent behavior suggests that it is necessary to explore in more detail the effects of biochar on forages of different nutritive value.

The biochar source and inclusion level interaction observed on VFA production, acetic acid and *iso*-butyric acid proportions and H_2_ generated was due to the AFB at 10%, no differences were observed among the other treatments, except for valeric acid. This is the first report of two biochars sources at two inclusion levels; in the previous works only different biochar sources or different biochar inclusion levels had been evaluated. However, biochar sources (Cabeza et al., 2018; McFarlane et al., 2017) and biochar inclusion levels (Saleem et al., 2018) were also reported to affect VFA production and individual VFA profile (Cabeza et al., 2018; McFarlane et al., 2017). Contrasting fermentation extent or profile may be due to differences in the biomasses used to produce biochar and pyrolysis conditions, resulting into biochars with different biochemical and structural properties. This will definitely influence characteristics as surface area, the pH_PZC_, and electroconductivity and thus affect the rumen microbial population, with impact on the multi-species biofilm formed and, ultimately, on the ruminal fermentation extent and profile.

## Conclusions

This study assessed, for the first time, the effects of two biochar sources (AFB and PPB) supplemented at two levels (5 and 10%, DM basis) added to two basal substrates (HS and CS) on CH_4_ production and major rumen fermentation parameters. Biochar supplementation, regardless of the source and level, increased CH_4_ production and modified the fermentation pattern. Nevertheless, the biochar source and inclusion level lead to similar effects on rumen fermentation parameters after 24-h incubation, except for VFA production and H_2_ produced and consumed. The similar behavior of the two different biochars suggests that other characteristics besides surface area and pyrolysis temperature may have important effects on the rumen fermentation pattern modulation, namely on CH_4_ production and yield. Considering that the use of biochars as a feed supplement for decreasing CH_4_ is still an emergent research topic, we believe that this work will add relevant information to the state of the art, by providing a systematic in-vitro comparison between two different biochars in controlled conditions. This work will also certainly impact on future studies that are still needed to clarify these complex interactions between biochar and the rumen ecosystem.

##  Supplemental Information

10.7717/peerj.9488/supp-1 Data S1 Raw data of all the experiments performed

## Additional Information and Declarations

### Data Availability

The following information was supplied regarding data availability: The raw measurements of all experiments are available in the Supplemental File.
